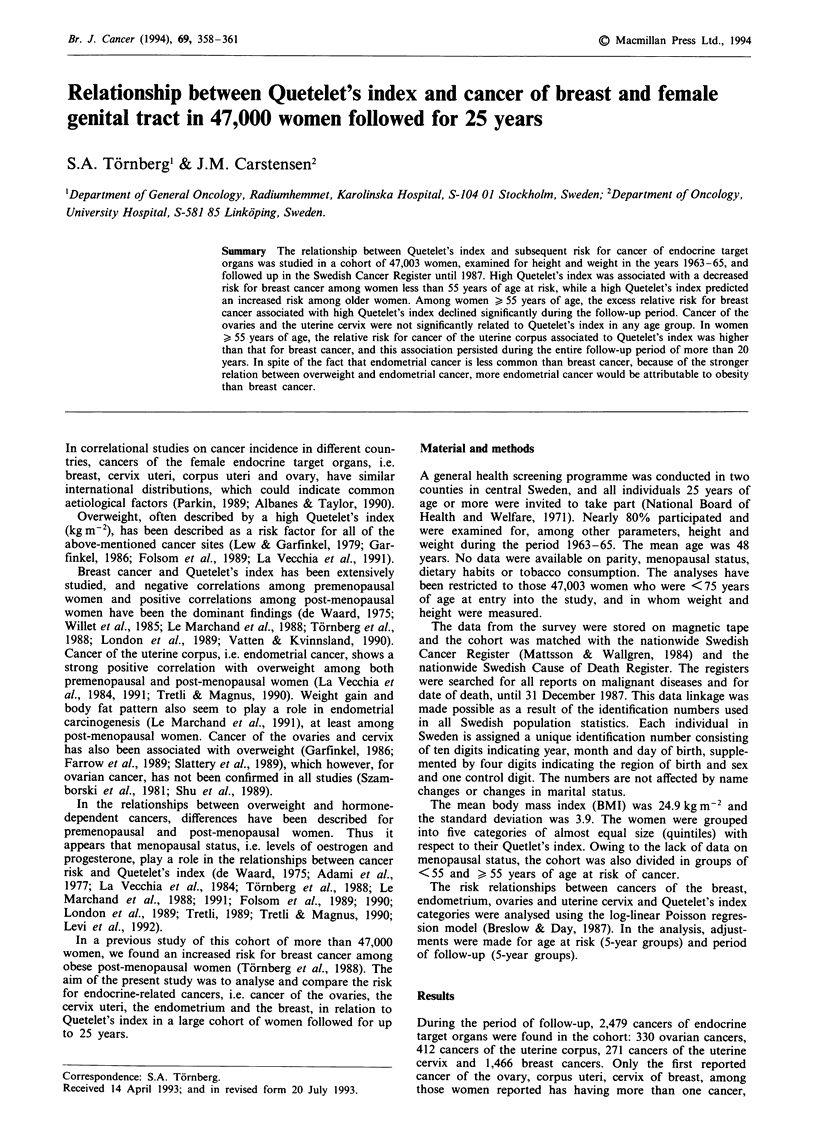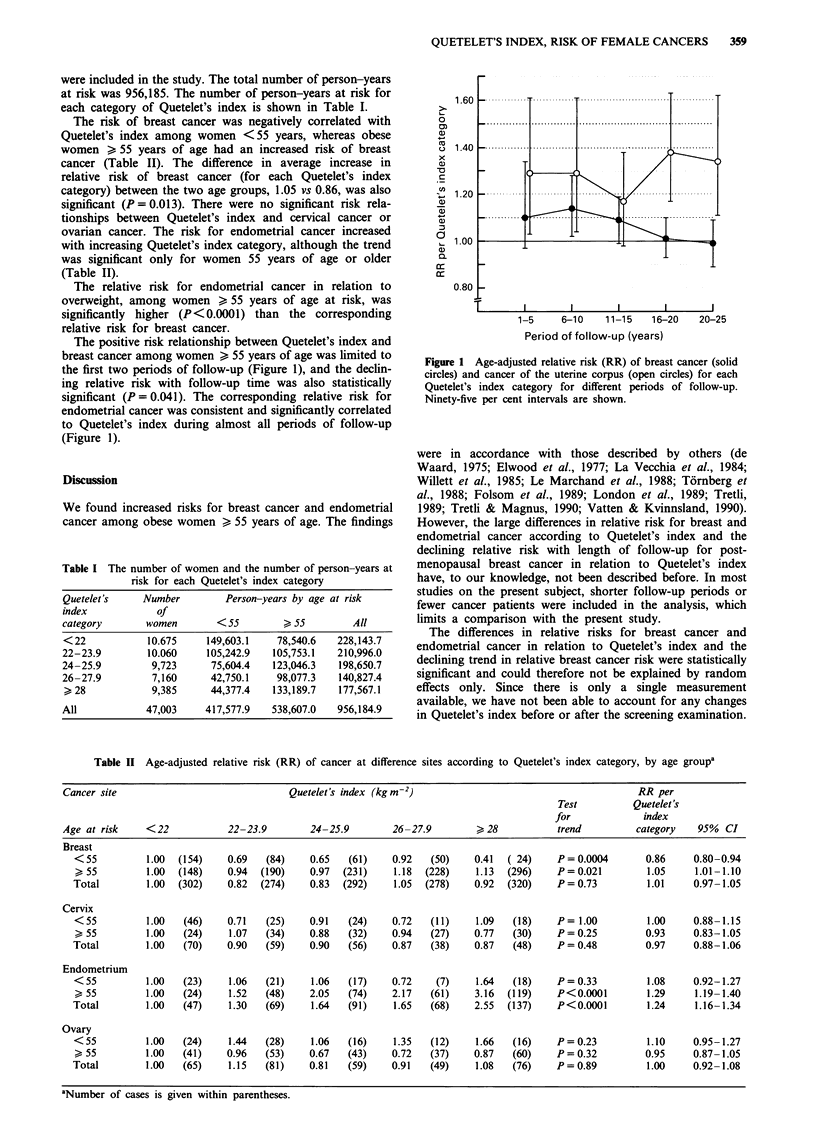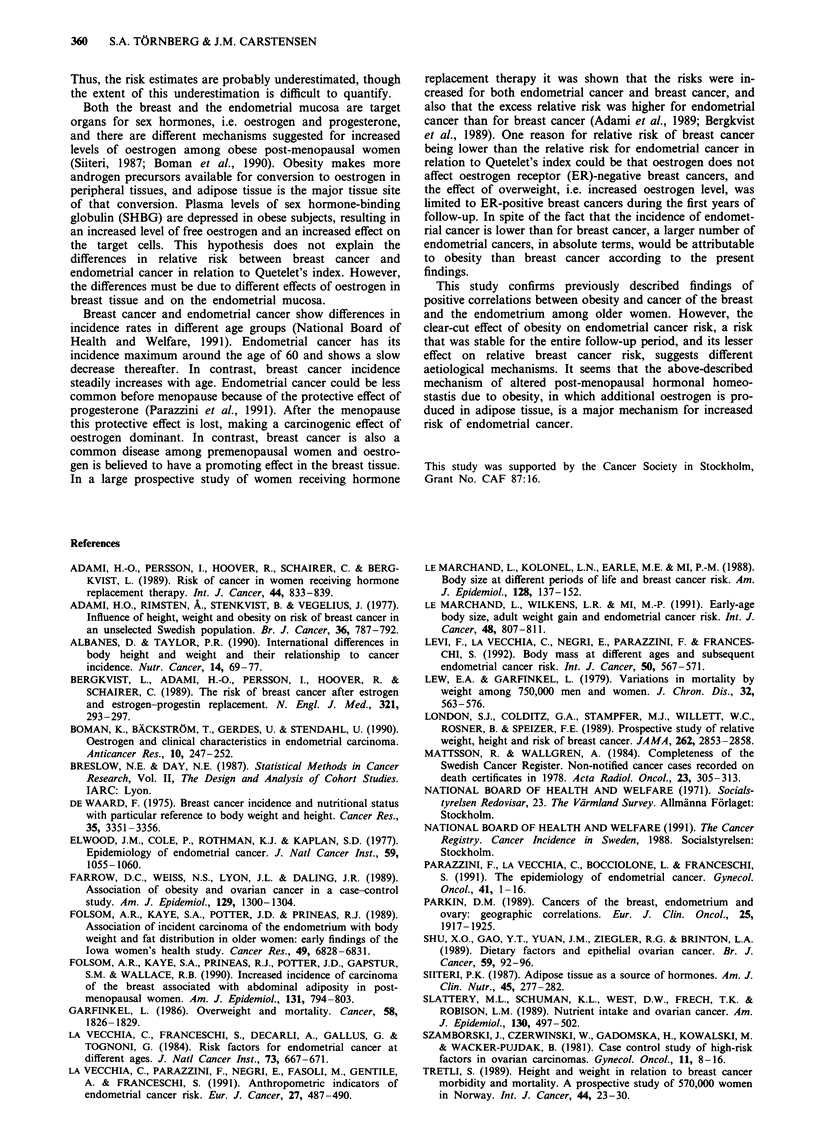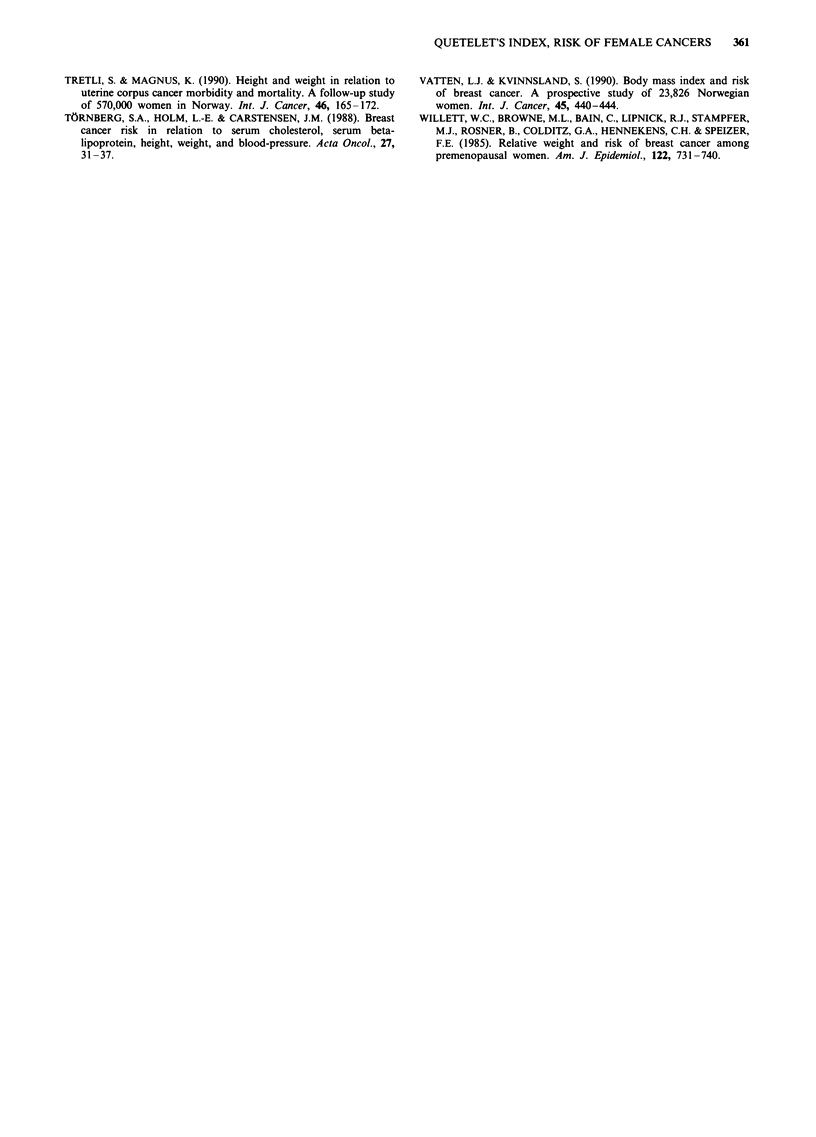# Relationship between Quetelet's index and cancer of breast and female genital tract in 47,000 women followed for 25 years.

**DOI:** 10.1038/bjc.1994.65

**Published:** 1994-02

**Authors:** S. A. Törnberg, J. M. Carstensen

**Affiliations:** Department of General Oncology, Radiumhemmet, Karolinska Hospital, Stockholm, Sweden.

## Abstract

The relationship between Quetelet's index and subsequent risk for cancer of endocrine target organs was studied in a cohort of 47,003 women, examined for height and weight in the years 1963-65, and followed up in the Swedish Cancer Register until 1987. High Quetelet's index was associated with a decreased risk for breast cancer among women less than 55 years of age at risk, while a high Quetelet's index predicted an increased risk among older women. Among women > or = 55 years of age, the excess relative risk for breast cancer associated with high Quetelet's index declined significantly during the follow-up period. Cancer of the ovaries and the uterine cervix were not significantly related to Quetelet's index in any age group. In women > or = 55 years of age, the relative risk for cancer of the uterine corpus associated to Quetelet's index was higher than that for breast cancer, and this association persisted during the entire follow-up period of more than 20 years. In spite of the fact that endometrial cancer is less common than breast cancer, because of the stronger relation between overweight and endometrial cancer, more endometrial cancer would be attributable to obesity than breast cancer.


Br. J. Cancer (1994), 69, 358-361
© Macmillan Press Ltd., 1994

### Relationship between Quetelet's index and cancer of breast and female genital tract in 47,000 women followed for 25 years

S.A. Törnberg[1] & J.M. Carstensen[2]

[1]*Department of General Oncology, Radiumhemmet, Karolinska Hospital, S-104 01 Stockholm, Sweden;* [2]*Department of Oncology, University Hospital, S-581 85 Linköping, Sweden.*

**Summary** The relationship between Quetelet's index and subsequent risk for cancer of endocrine target organs was studied in a cohort of 47,003 women, examined for height and weight in the years 1963-65, and followed up in the Swedish Cancer Register until 1987. High Quetelet's index was associated with a decreased risk for breast cancer among women less than 55 years of age at risk, while a high Quetelet's index predicted an increased risk among older women. Among women $\geqslant 55$ years of age, the excess relative risk for breast cancer associated with high Quetelet's index declined significantly during the follow-up period. Cancer of the ovaries and the uterine cervix were not significantly related to Quetelet's index in any age group. In women $\geqslant 55$ years of age, the relative risk for cancer of the uterine corpus associated to Quetelet's index was higher than that for breast cancer, and this association persisted during the entire follow-up period of more than 20 years. In spite of the fact that endometrial cancer is less common than breast cancer, because of the stronger relation between overweight and endometrial cancer, more endometrial cancer would be attributable to obesity than breast cancer.

In correlational studies on cancer incidence in different countries, cancers of the female endocrine target organs, i.e. breast, cervix uteri, corpus uteri and ovary, have similar international distributions, which could indicate common aetiological factors (Parkin, 1989; Albanes & Taylor, 1990).

Overweight, often described by a high Quetelet's index ($kg\,m^{-2}$), has been described as a risk factor for all of the above-mentioned cancer sites (Lew & Garfinkel, 1979; Garfinkel, 1986; Folsom *et al.*, 1989; La Vecchia *et al.*, 1991).

Breast cancer and Quetelet's index has been extensively studied, and negative correlations among premenopausal women and positive correlations among post-menopausal women have been the dominant findings (de Waard, 1975; Willet *et al.*, 1985; Le Marchand *et al.*, 1988; Törnberg *et al.*, 1988; London *et al.*, 1989; Vatten & Kvinnsland, 1990). Cancer of the uterine corpus, i.e. endometrial cancer, shows a strong positive correlation with overweight among both premenopausal and post-menopausal women (La Vecchia *et al.*, 1984, 1991; Tretli & Magnus, 1990). Weight gain and body fat pattern also seem to play a role in endometrial carcinogenesis (Le Marchand *et al.*, 1991), at least among post-menopausal women. Cancer of the ovaries and cervix has also been associated with overweight (Garfinkel, 1986; Farrow *et al.*, 1989; Slattery *et al.*, 1989), which however, for ovarian cancer, has not been confirmed in all studies (Szamborski *et al.*, 1981; Shu *et al.*, 1989).

In the relationships between overweight and hormone-dependent cancers, differences have been described for premenopausal and post-menopausal women. Thus it appears that menopausal status, i.e. levels of oestrogen and progesterone, play a role in the relationships between cancer risk and Quetelet's index (de Waard, 1975; Adami *et al.*, 1977; La Vecchia *et al.*, 1984; Törnberg *et al.*, 1988; Le Marchand *et al.*, 1988; 1991; Folsom *et al.*, 1989; 1990; London *et al.*, 1989; Tretli, 1989; Tretli & Magnus, 1990; Levi *et al.*, 1992).

In a previous study of this cohort of more than 47,000 women, we found an increased risk for breast cancer among obese post-menopausal women (Törnberg *et al.*, 1988). The aim of the present study was to analyse and compare the risk for endocrine-related cancers, i.e. cancer of the ovaries, the cervix uteri, the endometrium and the breast, in relation to Quetelet's index in a large cohort of women followed for up to 25 years.

Correspondence: S.A. Törnberg.
Received 14 April 1993; and in revised form 20 July 1993.

### Material and methods

A general health screening programme was conducted in two counties in central Sweden, and all individuals 25 years of age or more were invited to take part (National Board of Health and Welfare, 1971). Nearly 80% participated and were examined for, among other parameters, height and weight during the period 1963-65. The mean age was 48 years. No data were available on parity, menopausal status, dietary habits or tobacco consumption. The analyses have been restricted to those 47,003 women who were $<75$ years of age at entry into the study, and in whom weight and height were measured.

The data from the survey were stored on magnetic tape and the cohort was matched with the nationwide Swedish Cancer Register (Mattsson & Wallgren, 1984) and the nationwide Swedish Cause of Death Register. The registers were searched for all reports on malignant diseases and for date of death, until 31 December 1987. This data linkage was made possible as a result of the identification numbers used in all Swedish population statistics. Each individual in Sweden is assigned a unique identification number consisting of ten digits indicating year, month and day of birth, supplemented by four digits indicating the region of birth and sex and one control digit. The numbers are not affected by name changes or changes in marital status.

The mean body mass index (BMI) was $24.9\,kg\,m^{-2}$ and the standard deviation was 3.9. The women were grouped into five categories of almost equal size (quintiles) with respect to their Quetlet's index. Owing to the lack of data on menopausal status, the cohort was also divided in groups of $<55$ and $\geqslant 55$ years of age at risk of cancer.

The risk relationships between cancers of the breast, endometrium, ovaries and uterine cervix and Quetelet's index categories were analysed using the log-linear Poisson regression model (Breslow & Day, 1987). In the analysis, adjustments were made for age at risk (5-year groups) and period of follow-up (5-year groups).

### Results

During the period of follow-up, 2,479 cancers of endocrine target organs were found in the cohort: 330 ovarian cancers, 412 cancers of the uterine corpus, 271 cancers of the uterine cervix and 1,466 breast cancers. Only the first reported cancer of the ovary, corpus uteri, cervix of breast, among those women reported has having more than one cancer, were included in the study. The total number of person–years at risk was 956,185. The number of person–years at risk for each category of Quetelet's index is shown in Table I.

The risk of breast cancer was negatively correlated with Quetelet's index among women <55 years, whereas obese women ⩾55 years of age had an increased risk of breast cancer (Table II). The difference in average increase in relative risk of breast cancer (for each Quetelet's index category) between the two age groups, 1.05 *vs* 0.86, was also significant ($P = 0.013$). There were no significant risk relationships between Quetelet's index and cervical cancer or ovarian cancer. The risk for endometrial cancer increased with increasing Quetelet's index category, although the trend was significant only for women 55 years of age or older (Table II).

The relative risk for endometrial cancer in relation to overweight, among women ⩾55 years of age at risk, was significantly higher ($P < 0.0001$) than the corresponding relative risk for breast cancer.

The positive risk relationship between Quetelet's index and breast cancer among women ⩾55 years of age was limited to the first two periods of follow-up (Figure 1), and the declining relative risk with follow-up time was also statistically significant ($P = 0.041$). The corresponding relative risk for endometrial cancer was consistent and significantly correlated to Quetelet's index during almost all periods of follow-up (Figure 1).

### Discussion

We found increased risks for breast cancer and endometrial cancer among obese women ⩾55 years of age. The findings were in accordance with those described by others (de Waard, 1975; Elwood *et al.*, 1977; La Vecchia *et al.*, 1984; Willett *et al.*, 1985; Le Marchand *et al.*, 1988; Törnberg *et al.*, 1988; Folsom *et al.*, 1989; London *et al.*, 1989; Tretli, 1989; Tretli & Magnus, 1990; Vatten & Kvinnsland, 1990). However, the large differences in relative risk for breast and endometrial cancer according to Quetelet's index and the declining relative risk with length of follow-up for post-menopausal breast cancer in relation to Quetelet's index have, to our knowledge, not been described before. In most studies on the present subject, shorter follow-up periods or fewer cancer patients were included in the analysis, which limits a comparison with the present study.

The differences in relative risks for breast cancer and endometrial cancer in relation to Quetelet's index and the declining trend in relative breast cancer risk were statistically significant and could therefore not be explained by random effects only. Since there is only a single measurement available, we have not been able to account for any changes in Quetelet's index before or after the screening examination.

**Figure 1** Age-adjusted relative risk (RR) of breast cancer (solid circles) and cancer of the uterine corpus (open circles) for each Quetelet's index category for different periods of follow-up. Ninety-five per cent intervals are shown.

**Table I** The number of women and the number of person–years at risk for each Quetelet's index category

| *Quetelet's index category* | *Number of women* | *Person–years by age at risk* <55 | ⩾55 | All |
|---|---|---|---|---|
| <22 | 10.675 | 149,603.1 | 78,540.6 | 228,143.7 |
| 22–23.9 | 10.060 | 105,242.9 | 105,753.1 | 210,996.0 |
| 24–25.9 | 9,723 | 75,604.4 | 123,046.3 | 198,650.7 |
| 26–27.9 | 7,160 | 42,750.1 | 98,077.3 | 140,827.4 |
| ⩾28 | 9,385 | 44,377.4 | 133,189.7 | 177,567.1 |
| All | 47,003 | 417,577.9 | 538,607.0 | 956,184.9 |

**Table II** Age-adjusted relative risk (RR) of cancer at difference sites according to Quetelet's index category, by age group[a]

| *Cancer site* / *Age at risk* | *Quetelet's index* ($kg\,m^{-2}$) <22 | 22–23.9 | 24–25.9 | 26–27.9 | ⩾28 | *Test for trend* | *RR per Quetelet's index category* | *95% CI* |
|---|---|---|---|---|---|---|---|---|
| Breast | | | | | | | | |
| <55 | 1.00 (154) | 0.69 (84) | 0.65 (61) | 0.92 (50) | 0.41 ( 24) | $P = 0.0004$ | 0.86 | 0.80–0.94 |
| ⩾55 | 1.00 (148) | 0.94 (190) | 0.97 (231) | 1.18 (228) | 1.13 (296) | $P = 0.021$ | 1.05 | 1.01–1.10 |
| Total | 1.00 (302) | 0.82 (274) | 0.83 (292) | 1.05 (278) | 0.92 (320) | $P = 0.73$ | 1.01 | 0.97–1.05 |
| Cervix | | | | | | | | |
| <55 | 1.00 (46) | 0.71 (25) | 0.91 (24) | 0.72 (11) | 1.09 (18) | $P = 1.00$ | 1.00 | 0.88–1.15 |
| ⩾55 | 1.00 (24) | 1.07 (34) | 0.88 (32) | 0.94 (27) | 0.77 (30) | $P = 0.25$ | 0.93 | 0.83–1.05 |
| Total | 1.00 (70) | 0.90 (59) | 0.90 (56) | 0.87 (38) | 0.87 (48) | $P = 0.48$ | 0.97 | 0.88–1.06 |
| Endometrium | | | | | | | | |
| <55 | 1.00 (23) | 1.06 (21) | 1.06 (17) | 0.72 (7) | 1.64 (18) | $P = 0.33$ | 1.08 | 0.92–1.27 |
| ⩾55 | 1.00 (24) | 1.52 (48) | 2.05 (74) | 2.17 (61) | 3.16 (119) | $P < 0.0001$ | 1.29 | 1.19–1.40 |
| Total | 1.00 (47) | 1.30 (69) | 1.64 (91) | 1.65 (68) | 2.55 (137) | $P < 0.0001$ | 1.24 | 1.16–1.34 |
| Ovary | | | | | | | | |
| <55 | 1.00 (24) | 1.44 (28) | 1.06 (16) | 1.35 (12) | 1.66 (16) | $P = 0.23$ | 1.10 | 0.95–1.27 |
| ⩾55 | 1.00 (41) | 0.96 (53) | 0.67 (43) | 0.72 (37) | 0.87 (60) | $P = 0.32$ | 0.95 | 0.87–1.05 |
| Total | 1.00 (65) | 1.15 (81) | 0.81 (59) | 0.91 (49) | 1.08 (76) | $P = 0.89$ | 1.00 | 0.92–1.08 |

[a]Number of cases is given within parentheses.

Thus, the risk estimates are probably underestimated, though the extent of this underestimation is difficult to quantify.

Both the breast and the endometrial mucosa are target organs for sex hormones, i.e. oestrogen and progesterone, and there are different mechanisms suggested for increased levels of oestrogen among obese post-menopausal women (Siiteri, 1987; Boman *et al*., 1990). Obesity makes more androgen precursors available for conversion to oestrogen in peripheral tissues, and adipose tissue is the major tissue site of that conversion. Plasma levels of sex hormone-binding globulin (SHBG) are depressed in obese subjects, resulting in an increased level of free oestrogen and an increased effect on the target cells. This hypothesis does not explain the differences in relative risk between breast cancer and endometrial cancer in relation to Quetelet's index. However, the differences must be due to different effects of oestrogen in breast tissue and on the endometrial mucosa.

Breast cancer and endometrial cancer show differences in incidence rates in different age groups (National Board of Health and Welfare, 1991). Endometrial cancer has its incidence maximum around the age of 60 and shows a slow decrease thereafter. In contrast, breast cancer incidence steadily increases with age. Endometrial cancer could be less common before menopause because of the protective effect of progesterone (Parazzini *et al*., 1991). After the menopause this protective effect is lost, making a carcinogenic effect of oestrogen dominant. In contrast, breast cancer is also a common disease among premenopausal women and oestrogen is believed to have a promoting effect in the breast tissue. In a large prospective study of women receiving hormone replacement therapy it was shown that the risks were increased for both endometrial cancer and breast cancer, and also that the excess relative risk was higher for endometrial cancer than for breast cancer (Adami *et al*., 1989; Bergkvist *et al*., 1989). One reason for relative risk of breast cancer being lower than the relative risk for endometrial cancer in relation to Quetelet's index could be that oestrogen does not affect oestrogen receptor (ER)-negative breast cancers, and the effect of overweight, i.e. increased oestrogen level, was limited to ER-positive breast cancers during the first years of follow-up. In spite of the fact that the incidence of endometrial cancer is lower than for breast cancer, a larger number of endometrial cancers, in absolute terms, would be attributable to obesity than breast cancer according to the present findings.

This study confirms previously described findings of positive correlations between obesity and cancer of the breast and the endometrium among older women. However, the clear-cut effect of obesity on endometrial cancer risk, a risk that was stable for the entire follow-up period, and its lesser effect on relative breast cancer risk, suggests different aetiological mechanisms. It seems that the above-described mechanism of altered post-menopausal hormonal homeostastis due to obesity, in which additional oestrogen is produced in adipose tissue, is a major mechanism for increased risk of endometrial cancer.

This study was supported by the Cancer Society in Stockholm, Grant No. CAF 87:16.

### References

ADAMI, H.-O., PERSSON, I., HOOVER, R., SCHAIRER, C. & BERGKVIST, L. (1989). Risk of cancer in women receiving hormone replacement therapy. *Int. J. Cancer*, **44**, 833–839.

ADAMI, H.O., RIMSTEN, Å., STENKVIST, B. & VEGELIUS, J. (1977). Influence of height, weight and obesity on risk of breast cancer in an unselected Swedish population. *Br. J. Cancer*, **36**, 787–792.

ALBANES, D. & TAYLOR, P.R. (1990). International differences in body height and weight and their relationship to cancer incidence. *Nutr. Cancer*, **14**, 69–77.

BERGKVIST, L., ADAMI, H.-O., PERSSON, I., HOOVER, R. & SCHAIRER, C. (1989). The risk of breast cancer after estrogen and estrogen–progestin replacement. *N. Engl. J. Med*., **321**, 293–297.

BOMAN, K., BÄCKSTRÖM, T., GERDES, U. & STENDAHL, U. (1990). Oestrogen and clinical characteristics in endometrial carcinoma. *Anticancer Res*., **10**, 247–252.

BRESLOW, N.E. & DAY, N.E. (1987). *Statistical Methods in Cancer Research*, Vol. II, *The Design and Analysis of Cohort Studies*. IARC: Lyon.

DE WAARD, F. (1975). Breast cancer incidence and nutritional status with particular reference to body weight and height. *Cancer Res*., **35**, 3351–3356.

ELWOOD, J.M., COLE, P., ROTHMAN, K.J. & KAPLAN, S.D. (1977). Epidemiology of endometrial cancer. *J. Natl Cancer Inst*., **59**, 1055–1060.

FARROW, D.C., WEISS, N.S., LYON, J.L. & DALING, J.R. (1989). Association of obesity and ovarian cancer in a case–control study. *Am. J. Epidemiol*., **129**, 1300–1304.

FOLSOM, A.R., KAYE, S.A., POTTER, J.D. & PRINEAS, R.J. (1989). Association of incident carcinoma of the endometrium with body weight and fat distribution in older women: early findings of the Iowa women's health study. *Cancer Res*., **49**, 6828–6831.

FOLSOM, A.R., KAYE, S.A., PRINEAS, R.J., POTTER, J.D., GAPSTUR, S.M. & WALLACE, R.B. (1990). Increased incidence of carcinoma of the breast associated with abdominal adiposity in post-menopausal women. *Am. J. Epidemiol*., **131**, 794–803.

GARFINKEL, L. (1986). Overweight and mortality. *Cancer*, **58**, 1826–1829.

LA VECCHIA, C., FRANCESCHI, S., DECARLI, A., GALLUS, G. & TOGNONI, G. (1984). Risk factors for endometrial cancer at different ages. *J. Natl Cancer Inst*., **73**, 667–671.

LA VECCHIA, C., PARAZZINI, F., NEGRI, E., FASOLI, M., GENTILE, A. & FRANCESCHI, S. (1991). Anthropometric indicators of endometrial cancer risk. *Eur. J. Cancer*, **27**, 487–490.

LE MARCHAND, L., KOLONEL, L.N., EARLE, M.E. & MI, P.-M. (1988). Body size at different periods of life and breast cancer risk. *Am. J. Epidemiol*., **128**, 137–152.

LE MARCHAND, L., WILKENS, L.R. & MI, M.-P. (1991). Early-age body size, adult weight gain and endometrial cancer risk. *Int. J. Cancer*, **48**, 807–811.

LEVI, F., LA VECCHIA, C., NEGRI, E., PARAZZINI, F. & FRANCESCHI, S. (1992). Body mass at different ages and subsequent endometrial cancer risk. *Int. J. Cancer*, **50**, 567–571.

LEW, E.A. & GARFINKEL, L. (1979). Variations in mortality by weight among 750,000 men and women. *J. Chron. Dis*., **32**, 563–576.

LONDON, S.J., COLDITZ, G.A., STAMPFER, M.J., WILLETT, W.C., ROSNER, B. & SPEIZER, F.E. (1989). Prospective study of relative weight, height and risk of breast cancer. *JAMA*, **262**, 2853–2858.

MATTSSON, R. & WALLGREN, A. (1984). Completeness of the Swedish Cancer Register. Non-notified cancer cases recorded on death certificates in 1978. *Acta Radiol. Oncol*., **23**, 305–313.

NATIONAL BOARD OF HEALTH AND WELFARE (1971). *Socialstyrelsen Redovisar*, 23. *The Värmland Survey*. Allmänna Förlaget: Stockholm.

NATIONAL BOARD OF HEALTH AND WELFARE (1991). *The Cancer Registry. Cancer Incidence in Sweden*, 1988. Socialstyrelsen: Stockholm.

PARAZZINI, F., LA VECCHIA, C., BOCCIOLONE, L. & FRANCESCHI, S. (1991). The epidemiology of endometrial cancer. *Gynecol. Oncol*., **41**, 1–16.

PARKIN, D.M. (1989). Cancers of the breast, endometrium and ovary: geographic correlations. *Eur. J. Clin. Oncol*., **25**, 1917–1925.

SHU, X.O., GAO, Y.T., YUAN, J.M., ZIEGLER, R.G. & BRINTON, L.A. (1989). Dietary factors and epithelial ovarian cancer. *Br. J. Cancer*, **59**, 92–96.

SIITERI, P.K. (1987). Adipose tissue as a source of hormones. *Am. J. Clin. Nutr*., **45**, 277–282.

SLATTERY, M.L., SCHUMAN, K.L., WEST, D.W., FRECH, T.K. & ROBISON, L.M. (1989). Nutrient intake and ovarian cancer. *Am. J. Epidemiol*., **130**, 497–502.

SZAMBORSKI, J., CZERWINSKI, W., GADOMSKA, H., KOWALSKI, M. & WACKER-PUJDAK, B. (1981). Case control study of high-risk factors in ovarian carcinomas. *Gynecol. Oncol*., **11**, 8–16.

TRETLI, S. (1989). Height and weight in relation to breast cancer morbidity and mortality. A prospective study of 570,000 women in Norway. *Int. J. Cancer*, **44**, 23–30.

TRETLI, S. & MAGNUS, K. (1990). Height and weight in relation to uterine corpus cancer morbidity and mortality. A follow-up study of 570,000 women in Norway. *Int. J. Cancer*, **46**, 165–172.

TÖRNBERG, S.A., HOLM, L.-E. & CARSTENSEN, J.M. (1988). Breast cancer risk in relation to serum cholesterol, serum beta-lipoprotein, height, weight, and blood-pressure. *Acta Oncol.*, **27**, 31–37.

VATTEN, L.J. & KVINNSLAND, S. (1990). Body mass index and risk of breast cancer. A prospective study of 23,826 Norwegian women. *Int. J. Cancer*, **45**, 440–444.

WILLETT, W.C., BROWNE, M.L., BAIN, C., LIPNICK, R.J., STAMPFER, M.J., ROSNER, B., COLDITZ, G.A., HENNEKENS, C.H. & SPEIZER, F.E. (1985). Relative weight and risk of breast cancer among premenopausal women. *Am. J. Epidemiol.*, **122**, 731–740.